# Candidate gene based association mapping in *Fusarium culmorum* for field quantitative pathogenicity and mycotoxin production in wheat

**DOI:** 10.1186/s12863-017-0511-9

**Published:** 2017-05-19

**Authors:** Valheria Castiblanco, Jose J. Marulanda, Tobias Würschum, Thomas Miedaner

**Affiliations:** 1 State Plant Breeding Institute, University of Hohenheim, 70593 Stuttgart, Germany; 2 Institute of Plant Breeding, Seed Science and Population Genetics, University of Hohenheim, 79593 Stuttgart, Germany

**Keywords:** Aggressiveness, Quantitative pathogenicity, Fusarium head blight, Association mapping, Candidate genes

## Abstract

**Background:**

Quantitative traits are common in nature, but quantitative pathogenicity has received only little attention in phytopathology. In this study, we used 100 *Fusarium culmorum* isolates collected from natural field environments to assess their variation for two quantitative traits, aggressiveness and deoxynivalenol (DON) production on wheat plants grown in four different field environments (location-year combinations). Seventeen *Fusarium graminearum* pathogenicity candidate genes were assessed for their effect on the aggressiveness and DON production of *F. culmorum* under field conditions.

**Results:**

For both traits, genotypic variance among isolates was high and significant while the isolate-by-environment interaction was also significant, amounting to approximately half of the genotypic variance. Among the studied candidate genes, the mitogen-activated protein kinase (MAPK) *HOG1* was found to be significantly associated with aggressiveness and deoxynivalenol (DON) production, explaining 10.29 and 6.05% of the genotypic variance, respectively**.**

**Conclusions:**

To the best of our knowledge, this is the first report of a protein kinase regulator explaining differences in field aggressiveness and mycotoxin production among individuals from natural populations of a plant pathogen.

**Electronic supplementary material:**

The online version of this article (doi:10.1186/s12863-017-0511-9) contains supplementary material, which is available to authorized users.

## Background

Quantitative traits are a key feature in nature [1]. They are controlled by many genes, each contributing with a small effect to the overall phenotypic expression of a trait. Surprisingly, quantitative traits of pathogenicity have received only little attention in fungal biology [2, 3]. The expression of quantitative pathogenicity is not only controlled by the pathogen and the host, but also by the environment and their interaction [3]. Association mapping employing mixed models is a common method to dissect the genetic architecture of quantitative traits. Originally designed for the analysis of human diseases, association mapping is now extensively used in plant genetic research [4] either as genome-wide association study (GWAS) using anonymous molecular markers distributed across the whole genome or as candidate gene association by studying single nucleotide polymorphisms within candidate genes. The genetic basis of both methods is a linkage disequilibrium (LD) between molecular polymorphisms and phenotypic traits [4]. Therefore, a sound phenotypic trait evaluation is urgently required. Moreover, proper assessment of quantitative pathogenicity in natural field habitats, which accounts for host-by-environment and pathogen-by-environment-interaction, is indispensable for predicting disease risk and design effective breeding programs for durable resistance.

While qualitative plant-pathogen molecular interactions, especially gene-for-gene interactions, have been widely studied, quantitative realtionships have received far less attention [3, 5]. It is known that genes associated with qualitative pathogenicity frequently encode secreted effectors, which disassemble the host defense response [6, 7]. From the point of view of the host, some hypotheses for explaining the molecular mechanisms that control quantitative disease resistance have been suggested, although the authors remark that this is a poorly understood field [5]. From the point of view of the pathogen, the current understanding of the molecular mechanisms that control quantitative disease pathogenicity is even more scarce. Plant-pathogen qualitative interactions are frequent in biotrophic and obligate pathogens, while hemibiotrophic/necrotrophic pathogens more often show quantitative interactions. It has been proposed that host range, life style and speciation of pathogens are shaped by the type of molecular communication with the host, e.g. the ability to rapidly evolve pathogen effector repertoires [8]. In this context, attempts to answer the following questions are highly relevant: Which are the key molecular players in the quantitative disease pathogenicity? Are they located in the first line of molecular communication with the host, as effectors which are secreted, or are they masters of the molecular regulatory cascades, as transcription factors? Furthermore, are there different allelic variants of those “key players” and what is their role in aggressiveness? *Fusarium culmorum* is a hemibiotroph, with a wide host range encompassing most of the cereals [9], which makes it a perfect model for the study of quantitative interactions.

Fusarium head blight (FHB) is a devastating disease of bread wheat (*Triticum aestivum* L.) and other small-grain cereals worldwide [10]. It leads to significant losses not only in terms of yield but also quality, because of the contamination of kernels by mycotoxins, which pose a significant risk to human and animal health [11]. Isolates causing FHB can be classified by their production of type B trichothecenes, including nivalenol (NIV), deoxynivalenol (DON) and its acetylated forms 3-acetyl-deoxynivalenol (3-ADON) and 15-acetyl-deoxynivalenol (15-ADON), as well as estrogen analogues like zearalenone (ZEA) and other mycotoxins [12]. DON is the predominant and economically most important trichothecene detected in cereals in Europe [12]. Therefore, the European Union has set a limit for DON in unprocessed bread wheat for human consumption of 1.25 mg/kg [13]. Various *Fusarium* species have been reported as causal agents of FHB, among them the haploid ascomycetes *Fusarium graminearum* and *Fusarium culmorum* are two of the most important. *F. culmorum* has been traditionally related with FHB epidemics in the Mediterranean region [14–16] as well as northern, central and western Europe [17–20]. In contrast to *F. graminearum*, which has been extensively studied and whose full genome sequence has been published [21], a draft of *F. culmorum* genome was just recently released [22].

Aggressiveness, i.e. the quantitative pathogenicity as described by Van der Plank [23], and DON production are important determinants of parasitic fitness in *Fusarium* species [24, 25]. There is no generally recognized specific interaction between wheat genotypes and FHB causal agents [26, 27]. Resistant cultivars stay resistant even when challenged by highly aggressive isolates [28]. Aggressiveness is usually evaluated by directly measuring epidemic rates in this monocyclic disease. Higher aggressiveness is associated with faster symptom development and a larger amount of mycelium within the host tissue [29]. DON plays a key role in *F. graminearum* aggressiveness [30, 31] enabling faster pathogen spread from infected florets into the wheat rachis [32]. A distribution indicative of a quantitative inheritance was demonstrated in *F. graminearum* segregating progenies of three biparental crosses of genetically diverse isolates for both, aggressiveness and DON production [28, 33]. In contrast, the perfect stage (teleomorph) of *F. culmorum* is unknown, and therefore such studies could to date not be performed [34]. In order to understand the inheritance and the genetic control of aggressiveness in *F. culmorum*, the historically accumulated variation in natural populations can be exploited.

Thousands of genes have been characterized affecting host-fungus interaction in agricultural pathosystems, and many of them were found to have a pleiotropic effect [35, 36]. These experiments mainly worked with knock-out or deletion mutants, which are not adequate for explaining quantitative differences among isolates, because frequently the expression of aggressiveness is confounded with basic survival functions of the fungi in these studies. Candidate-gene association mapping appears as a promising and powerful tool to detect functional polymorphisms associated with differences in aggressiveness and DON production in *F. culmorum* populations.

To enhance our understanding of quantitative pathogenicity, we performed a candidate gene association mapping based on pathogenicity-related candidate genes that firstly, have been reported or predicted as related with quantitative variations in pathogenicity, but do not affect the survival of the fungus and secondly, play a role in the pathogenicity signaling cascade. Based on the sequenced *F. graminearum* genome, *F. culmorum* homologues for four transcription factors, eight signal transducers, two membrane receptors and three secreted proteins were sequenced. Using a natural population of 100 isolates of *F. culmorum* of diverse origin, we aimed to (i) determine the phenotypic variance for aggressiveness and DON production in replicated field experiments across two locations and 2 years with a moderately susceptible bread wheat cultivar as host, (ii) study sequences of the 17 candidate genes to identify nucleotide diversity within selected gene regions, (iii) test these quantitative trait nucleotides (QTNs) for their association with aggressiveness and DON production and estimate the proportion of genotypic variance explained by them.

## Methods

### Fungal materials and field trials

One hundred *F. culmorum* isolates from a previous diversity study [18] were phenotyped under field conditions for aggressiveness and deoxynivalenol (DON) production following a chessboard design described previously [37, 38]. In short, field plots were arranged such that each inoculated entry plot of bread wheat was surrounded by four border plots of a tall triticale cultivar (*x Triticosecale*) to minimize plot-by-plot interference. Fungal material consisted of four field populations (one from Russia and three from Germany), one transect population from Syria and one international collection (Additional file 1). The field populations were randomly collected isolates from symptom-bearing FHB infected heads from individual commercial wheat fields as described earlier [18]. All isolates were used as single-spore derived isolates.

Field experiments with these isolates were conducted during 2014 and 2015, and across two locations: Hohenheim (HOH, longitude 9° 11′ 23″ E, latitude 48° 42′ 54″ N, altitude 403 m) and Oberer Lindenhof (OLI, longitude 9° 18′ 17″, latitude 48° 28′ 25″ N, altitude 702 m) in Southwestern Germany. The experiments followed an incomplete block design (α design) with two replications. Experimental units were three-row plots (1.0 m long, 0.625 m wide), which were machine sown with 220 kernels per m^2^, a seeding rate that results in a homogeneous wheat stand. A moderately susceptible winter wheat cultivar was used as host (“Inspiration”, KWS LOCHOW GMBH, Bergen, Germany) with a FHB rating of 6 on the 1–9 scale, where 1 = without disease and 9 = fully infected.

Inoculum production was done in shaking cultures according to an existing procedure [39]. For inoculation, a dose of 100 ml suspension per square meter in a concentration of 2 × 10^5^ conidiospores ml^−1^ was sprayed onto wheat heads during full flowering. Inoculum for each isolate was sprayed on its corresponding plot, according to the randomization of the experiment design, using a hand atomizer with constant air pressure of 3 bar from a tractor to ensure full coverage of all heads of the plot with the same dosage. All plots flowered simultaneously, because only one homogeneous wheat cultivar was used. This allowed inoculation and ratings for all plots at the same dates per location. Contamination with natural inoculum was negligible as verified by the border plots which had always a FHB rating < 5%.

### Phenotyping aggressiveness and DON production

To assess aggressiveness of isolates, FHB symptoms were visually rated in each plot at least three times starting with the onset of symptom development, about 14 days after inoculation and was continued at 3- to 5-day intervals until the beginning of the yellow ripening stage. Rating was performed as percentage of infected spikelets per plot (0–100%). This reflects both the percentage of infected spikes per plot and the percentage of infected spikelets per spike in a single rating. The arithmetic mean of at least three ratings (i.e. the mean FHB ratings was used as aggressiveness trait).

To measure DON production, wheat plots were harvested by hand at full ripening, carefully threshed in a single-head thresher (Walter-Wintersteiger, Austria) and cleaned with reduced wind speed. The remaining fragments of glumes and rachis were manually picked out to retain highly shrunken kernels in the sample. Cleaned wheat grain was ground in a commercial laboratory mill with a sieve size of 1 mm. Later, the coarse meal was analyzed to quantify the amount of DON by a commercially available immunotest (R-Biopharm AG, Darmstadt, Germany).

### Phenotypic data analyses

Phenotypic data of aggressiveness and DON production were obtained from one experiment performed during 2 years and across two locations, yielding four test environments. Aggressiveness data and DON production were arcsin transformed to meet the required normal distribution and subjected to outlier detection with the method BH-MADR (Bonferroni–Holm with re-scaled MAD standardized residuals) suggested by Bernal-Vasquez et al. [40]. For the description of the model, the syntax suggested by Patterson [41] was followed, where fixed effects (E, R, I) appear before the colon and random effects (I × E, B) after the colon. In order to obtain best linear unbiased estimators (BLUE) of each isolate genotype, the phenotypic data were analyzed based on the following linear mixed model:

$$ \mathrm{E}+\mathrm{R}+\mathrm{I}:{\mathrm{I}}^{\times}\mathrm{E}+\mathrm{B}, $$

where E, R, I, and B denote environment, replication, isolate, and block or their interaction, respectively. Variance components were determined by the restricted maximum likelihood (REML) method. Significance of variance components was tested by model comparisons with likelihood ratio tests [42]. Heritability (*h* ^*2*^) was estimated as heritability on an entry-mean basis following the approach suggested by Piepho and Möhring [43]. All statistical analyses were performed using ASReml 3.0 [44] and R [45] combined with the graphical user interface RStudio.

### Gene selection and sequencing

Seventeen candidate genes with a confirmed or predicted role in pathogenesis of *Fusarium* spp., aggressiveness and/or trichothecene biosynthesis were used in our study (Table 1). Based on previous studies suggesting that genes associated with aggressiveness are involved in regulation or transport activities [37, 38], we focused mainly on those groups of genes, although few genes codifying for proteins located in membrane and secreted proteins were also included. The most variable regions of the selected genes were identified using BLAST analysis. Specific primers (Additional file 2) for amplification of those regions in each gene were designed using the software Primer3 - version 0.4.0 [46]. DNA extracted from each of the 100 isolates was used in a polymerase chain reaction (PCR) with the designed primers for each gene, following a standard protocol. Amplicons for each isolate and gene combination were sequenced once using the Sanger method. The sequences were aligned against the reference sequence of *F. graminearum* in the revised complete genome of the strain PH-1, RRes v4.0 [47], available within ENSEMBL fungi (http://fungi.ensembl.org) using the program MEGA6 [48] to identify single nucleotide polymorphisms (SNPs) among the 100 isolates. Polymorphisms that had more than 20% missing values or a minor allele frequency (MAF) of <5% were not considered for further analyses.

**Table 1:** Identity of candidate genes under study

Rresv4.0 annotation^a^	Gene ID	SNPs	Predicted/confirmed function
Genes encoding transcription factors			
FGRRES_12164	*FGP1*	0	Regulates pathogenicity, toxin synthesis and reproduction in *F. graminearum* [86]
FGRRES_00472	*SCH*	0	Regulates conidium size, stress responses and pathogenesis in *F. graminearum* [89]
FGRRES_06874	*TOP1*	0	Pathogenesis and sporulation in *F. graminearum* and *F. culmorum* [90]
FGRRES_08811	*EFTU*	1	Elongation factor 1α elicit an immune response in the host (Pathogen Associated Molecular Pattern, PAMP) and was identified as differentially secreted in the study of Rampitsch [61]
Genes encoding proteins involved in signal transduction			
FGRRES_06878	*CMK1*	1	Predicted virulence associated protein by Lysenko et al. [107], probable Cmk1/2 protein kinase type I [62]
FGRRES_16491	*STE11*	1	Belongs to MAPK module that regulates fungal development and pathogenicity in *F. graminearum* [93]
FGRRES_08531	*ERF2*	1	Associated with aggressiveness in the study of Talas et al. [37]
FGRRES_09612	*HOG1*	3	Regulates hyphal growth, stress responses and plant infection in *F. graminearum*. [92]
FGRRES_16251	*TRI6*	2	Global transcription regulator in *F. graminearum* associated with affected severity in *F. culmorum* [108, 109]
FGRRES_15765	*LAEA1*	0	Involved in control of secondary metabolism, sexual development and virulence in *F. graminearum* [87]
FGRRES_16620	*FLBA*	0	Involved in conidia production, sexual development, spore germination, mycotoxin production and virulence [88]
FGRRES_09614	*GPA*	0	Required for pathogenicity and normal growth [110]
Genes encoding membrane proteins			
FGRRES_09435	*SHO1*	0	Fungal development and pathogenicity [93]
FGRRES_05633	*MSB2*	3	Transmembrane sensor that regulates invasive growth and plant infection in fungi [93, 111, 112]
Genes encoding secreted proteins			
FGRRES_02342_M	*CUT*	17	Predicted cutinase, required to penetrate the host cuticle [61]
FGRRES_05906	*FGL1*	4	Secreted fungal effector lipase [113–115]
FGRRES_00838	*HSP70*	1	Belong to the family Hsp70 involved in heat-shock response and was found to be secreted differentially under pathogenicity conditions in *F. graminearum* by Rampitsch et al. [61]

For each selected gene, the number of single nucleotide polymorphisms (SNPs) detected with minor allele frequencies (MAF) >5% and function is reported

^a^The given ID (FGSG) is the entry number of the Rres v4.0 annotation *F. graminearum* genome database [47]

### Association mapping

Modified Rogers genetic distances (RD; Rogers [49]) based on 10 SSR markers were computed among all isolates under study, using the software *Plabsoft* [50]. Principal coordinate analysis (PCoA) was conducted on the Modified Rogers’s distances. Additionally, pairwise kinship coefficients [51] among individuals in the collection were estimated as 1 minus the modified Rogers’ distance [52]. A mixed linear model incorporating the BLUES obtained from the phenotypic analysis (Additional file 3), SNP’s information (Additional file 4), the three main principal coordinates as fixed effect and a kinship matrix for the random genotypic isolate effect was used to identify marker-trait associations [53]. The *p* values obtained from the one-degree-of-freedom score test were corrected for possible inflation [53]. The proportion of genotypic variance (ρ_G_) explained by each SNP was derived from the sums of squares of the SNP in a linear model using aggressiveness or DON production as dependent variables. As we found high multi-colinearity among SNPs within the candidate genes, only one SNP out of a group of highly linked SNPs (*HOG1–*380) was used in the linear model to assess the proportion of explained genotypic variance [54]. All calculations were done with the open-source statistical software R [45] including packages GenABEL [53] and APE [55]. The squared correlation coefficient (*r* ^2^) was used to estimate linkage disequilibrium (LD) between each pair of marker loci [56] using TASSEL [57].

## Results

### Phenotypic data

**Fig. 1:**
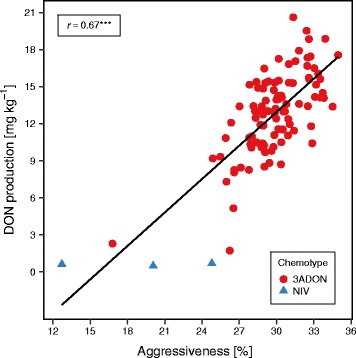
Relationship between mean aggressiveness and deoxynivalenol (DON) production across 100 *F. culmorum* isolates

**Fig. 2:**
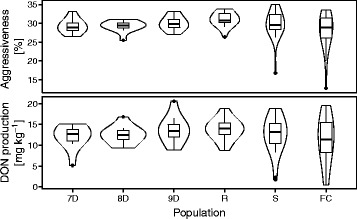
Violin boxplots of mean aggressiveness and deoxynivalenol (DON) production for populations under study after inoculation on bread wheat across four environments. Field populations consisted in Entringen [7D], Herrenberg [8D], Nufringen [9D] and Novgorod [R], one transect population from Syria [S], and the collection of 22 isolates of *Fusarium culmorum* [FC]. Horizontal line within boxes = median, • = outliers

All *F. culmorum* isolates successfully produced symptoms on the inoculated wheat spikes and differences among isolates were observed at all environments. The overall mean of aggressiveness was 15.97%, varying from a minimum of 4.2% for isolate FC60 and a maximum of 19.8% for the isolate S109. The overall mean for DON production was 12.98 mg kg^−1^ ranging from 0.7 mg kg^−1^ to 22.95 mg kg^−1^ for FC50 and 9D22, respectively. Nivalenol (NIV) chemotypes displayed always the lowest DON production values as well as lowest aggressiveness values. Significant correlation between aggressiveness and DON production was observed (*r* = 0.67, *p* < 0.001, Fig. 1). The overall entry-mean heritability was 0.87 for aggressiveness and 0.90 for DON production, illustrating a high relevance of genotypic variance. Accordingly, genotypic variances across all environments were significantly (*p* < 0.001) different from zero for both traits. Isolate-by-environment interaction was also significantly different from zero and about half that of the genotypic isolate variance (Table 2). The frequency distribution of aggressiveness and DON production was continuous and followed a normal distribution for both traits (Additional file 5). Variability within and among field populations, for aggressiveness and DON production, were observed. While the population from Russia produced on average the highest values for aggressiveness and DON production, the population 7D from Entringen, Germany, as well as the international collection had the lowest values for both traits (Fig. 2). Transect population from Syria displayed a large range for both trait values, almost the same range as displayed by the international collection (Fig. 2).

**Table 2:** Means, ranges and variance components of aggressiveness and deoxynivalenol (DON) production in two locations and 2 years

Parameter	Aggressiveness (%)	DON production
		(mg kg^−1^)
Means and ranges		
2014-HOH	28.80 (6.80–44.16)	14.75 (0.05–34.27)
2014-OLI	14.26 (3.50–30.50)	21.50 (0.19–55.99)
2015-HOH	8.98 (2.33–23.83)	9.05 (0.09–29.20)
2015-OLI	11.78 (1.00–25.00)	6.46 (0.11–24.96)
Combined	15.97 (1.0–44.16)	12.98 (0.05–55.99)
Variance components and heritabilities^a^		
${\sigma}_I^2$	1.15 × 10^–3 ***^	4.80 × 10^–3 ***^
${\sigma}_{I\times E}^2$	8.29 × 10^–4***^	1.84 × 10^–3 ***^
${\sigma}_e^2$	1.32 × 10^−3^	4.17 × 10^−3^
*h* ^*2*^	0.87	0.90

Ranges (in brackets), variance components for isolate $\left({\sigma}_I^2\right)$, isolate × environment interaction $\left({\sigma}_{I\times E}^2\right)$, error $\left({\sigma}_e^2\right)$, and entry-mean heritabilities (*h* ^*2*^)

*HOH* Hohenheim, *OLI* Oberer Lindenhof

^***^ Significant at *p* < 0.001

^a^Variance components and heritabilities calculated with arcsin transformed data

### Genotypic and molecular analysis

A principal coordinate analysis based on the modified Rogers’ distances among the 100 entries as revealed by SSR markers was performed [18]. The first three main coordinates explained 17, 10 and 10% of the molecular variance and were used to correct for population stratification in the subsequent association analysis (Additional file 6). In accordance with previous results, we observed two clusters setting Syrian samples apart from the German isolates, but not from the international collection.

**Fig. 3:**
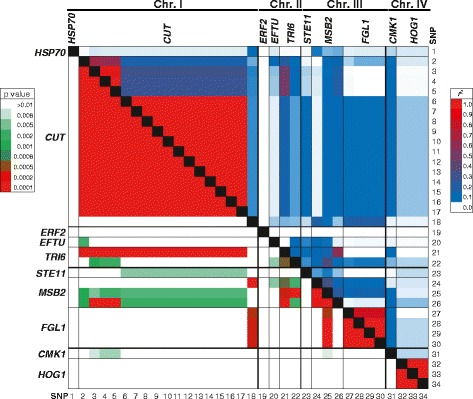
Pairwise linkage disequilibrium (LD) within and among 10 candidate genes based on 100 *Fusarium culmorum* isolates. LD measured as *r* ^*2*^ between all pairs of selected SNP loci (above diagonal) and significance (below diagonal). The horizontal and vertical lines separate the candidate genes and chromosomes

Analysis of the sequences of 17 genes for 100 *F. culmorum* isolates included in our study revealed 34 SNPs (Additional file 7) with a minor allele frequency higher than 5%, distributed in ten out of the 17 analyzed genes (Table 1). The gene displaying the highest number of SNPs was *CUT* with 17 SNPs, followed by *FGL1* with four SNPs. Different numbers of SNPs were found in genes with different function and cellular location of the encoded proteins. Genes encoding secreted proteins displayed the majority of SNPs, while no SNPs were found in any of the genes encoding transcription factors included in this study (*FGP1*, *SCH9*, *TOP1*). High LD was detected among SNPs within individual genes (Fig. 3) and within SNPs from two genes located on the same chromosome (*MSB2-FGL1*). Additionally, some SNPs in genes on different chromosomes showed high LD, for instance, the genes *TRI6/MSB2, TRI6/CUT, MSB2/CUT. HOG1* displayed the lowest level of LD with any of the other studied genes.

### Association analysis for aggressiveness and DON production

**Fig. 4:**
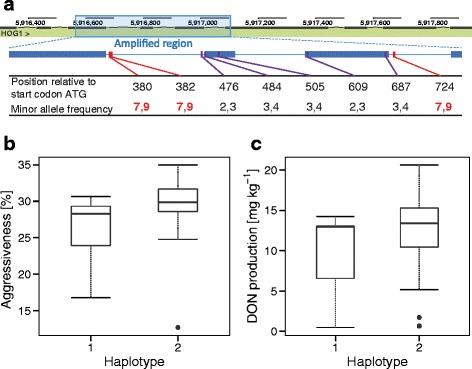
*HOG1* sequenced region, SNPs found and genetic effects of the two haplotypes. **a** Gene structure taken from ENSEMBL fungi database [116] modified to show *HOG1* sequenced region on chromosome four and all SNPs found. The location of the gene in *F. graminearum* genome is expressed in base pairs. Minor allele frequency expressed in percentage. Boxplots diagrams depicting the genetic effects of two *HOG1* haplotypes with significant associations to (**b**) aggressiveness and (**c**) DON production

Three SNPs closely linked to each other and located in *HOG1* were found to be significantly associated (*p* < 0.05) with aggressiveness and DON production. Given high collinearity, all three SNPs were analyzed as only one haplotype, which explains 10.29 and 6.05% of the genotypic variance of aggressiveness and DON production, respectively. Any haplotype was restricted to a single population, so no bias by population structure can be expected. Additionally, we used principal coordinate and kinship matrix analysis to correct for population structure. Associated polymorphisms are located within the gene at positions 380, 382 (intron 3) and 724 (intron 5) relative to the start codon (Fig. 4) and correspond to changes in base pairs C/T, C/T and G/A, respectively. Five more polymorphisms in introns and exons were found in *HOG1*, but were excluded from the analysis due to the minor allele frequency limit of 5%. Isolates having the most frequent *HOG1* haplotype expressed on average highest values for aggressiveness and DON production (Fig. 4). An association close to the significant threshold was also detected for one SNP in *TRI6* (TRI147, *p* = 0.057) with DON production and one insertion in *CUT* (CUT536 + 3, *p* = 0.07) with aggressiveness (Additional file 7). Both were excluded from further analysis because the *p* values were slightly higher than the threshold 0.05.

## Discussion

Given the economic and public health importance of Fusarium head blight [10, 35], the causal fungi have been under intensive investigation and became a model to study quantitative plant-microbe interactions. The release of the *F. graminearum* genome sequence in 2007 [21] motivated research on this pathogen using a wide variety of approaches to better understand the basis of pathogen biology. As a result, genes related to pathogenesis and other responses have been identified. Special attention has been given to genes encoding proteins of the secretome [58–61], kinome [62] and phosphatome [63]. However, little is known about which of those identified genes play an active role in explaining quantitative differences in aggressiveness among isolates occurring in natural populations, as the vast majority of studies have used only one *Fusarium* isolate. Furthermore, aggressiveness is a quantitative trait that is highly influenced by the environment. Thus, it is not surprising that candidate genes identified under controlled environments may not be relevant under field conditions [64–66]. In this study, we used 100 *F. culmorum* isolates collected from natural environments to evaluate the effect of previously identified candidate genes on aggressiveness and DON production across four field environments.

### Large genetic variation of isolates from individual fields for aggressiveness and DON production

Quantitative variation within natural field populations has previously only been reported for a few plant pathogens like *Rhynchosporium commune* [67], *Zymoseptoria tritici* [68–70] and the close relative of *Fusarium culmorum*, *F. graminearum* [71]. To our knowledge, this is the first study reporting variability in aggressiveness and DON production within *F. culmorum* isolates from natural field populations.

Both traits under study followed a continuous distribution, as expected for quantitative traits (Additional file 5). We observed a high correlation between DON production and aggressiveness (*r* = 0.67, *p* < 0.001, Fig. 1), which is consistent with a previous study using 42 *F. culmorum* isolates [72] and *F. graminearum* [71]. Even if the correlation cannot conclude causality of DON in aggressiveness [72], this evidence confirms an important role of DON in aggressiveness. Moreover, high infestation on the field is represented by high levels of DON contamination on harvested grain, despite the fact that highly aggressive isolates do not necessarily produce more DON per mycelium unit than less aggressive ones [72].

### Large isolate-by-environment interaction highlights the importance to study FHB using multi-environmental field trials

The genotypic variance is the component of phenotypic variability that could be exploited by selective forces [73, 74]. The observed genotypic variance, which corresponds to the isolate variance component in this study, was high and significant for both traits. In the light of evolution, our results suggest, that if aggressiveness increases the fitness of the population even during the saprophytic phases of the life cycle, the most aggressive genotypes will, with time and under a constant selection pressure, predominate in the population. However, the role of aggressiveness during the saprophytic phases of the life cycle are still to be studied.

Conducting experiments as multi-environment trials, using plots rather than single plants as experimental units and applying mixed models in the statistical analysis are standard practices in the study of quantitative traits of plants [75]. These approaches have only recently been applied to study quantitative plant-pathogen relationships [37, 38]. Here we report an isolate-by-environment interaction significantly different from zero and amounting to about half that of the isolate genotypic variance. These results are in accordance with previous studies [38, 76] and illustrate the relevance of methods which account for the isolate-by-environment interaction in the study of quantitative disease pathogenicity.

### High nucleotide variation found in candidate genes encoding secreted proteins

The *F. culmorum* draft genome sequence is currently under study, but gene annotation has not been completed yet [22]. Therefore, a wide array of information has been inferred from the well annotated reference genome sequence of its close relative *F. graminerum* [17, 77]. We designed primers for known genes in *F. graminearum* and used them successfully for amplification of all homologous genes in *F. culmorum* isolates. This further supports a high synteny and sequence homology among *F. culmorum* and *F. graminearum* genomes [78, 79].

The fact that the majority of SNPs were found in genes encoding secreted or membrane proteins, could be explained by their role in the molecular communication with the host and signal perception from the environment, which might be subject to positive selection pressure as widely reported [80–85]. By contrast, no informative SNPs were found in any of three out of four transcription factors included in this study (*FGP1*, *SCH9*, *TOP1*). This result and previous information concerning the function of those genes, suggested that they might be involved in the pleiotropic control of several basic physiological functions, among them pathogenicity [86–93].

High and significant LD was found among genes from different chromosomes, as for example *TRI6* and *CUT* (Fig. 3). Similar results were found in a study on *F. graminearum* reporting high levels of LD between genes of the TRI cluster and pathogenicity related candidate genes located on different chromosomes e.g. *TRI5/MetAP1*, *TRI10/MetAP1* [37]. Several hypotheses could explain these results: (i) simultaneous selection on the linked genes as a result of a common role in aggressiveness or a coordinated involvement in the same response pathway or (ii) low physical distance with other genes, which are responsible for the LD between different chromosomes.

### *HOG1*, a gene involved in osmotic and oxidative stress is associated with quantitative pathogenicity

*HOG1* was significantly associated with aggressiveness and DON production in our study. The mitogen-activated protein kinase (MAPK) *HOG1* is a core component of the high osmolarity glycerol (HOG) pathway and has been well characterized in *S. cerevisiae* [94]. MAPK pathways are three-tiered protein kinase modules that are present in all eukaryotic organisms and function in succession to transmit a variety of cellular signals [95]. Most fungal pathogens contain three MAPKs that are orthologues of the *S. cerevisiae* Fus3/Kss1, Slt2, and Hog1 MAPKs, and function in separate signaling cascades to regulate infection-related morphogenesis, cell wall remodeling, and high osmolarity stress response, respectively [96–99]. The first functional characterization of the kinome in a plant pathogenic fungi was developed using *Fusarium graminearum* as a model and generated deletion mutants for 96 protein kinase genes, out of them 42 kinase mutants were significantly reduced in virulence or non-pathogenic [62]. The *FgHOG1* is a core component of the HOG pathway in *F. graminearum*, which has been involved in the response to various environmental stresses [92]. Based on our results, we hypothesize that changes in *HOG1* regulation confer advantages in the response of *F. culmorum* to multiple stresses, especially to the osmotic and oxidative stresses resulting from the plant defense mechanisms. Our study further supports the observation of Talas et al. [38], suggesting that most of the genes associated with aggressiveness are involved in regulation or transport activities.

Quantitative traits are controlled by complex interactions of genes, and therefore single or few genes explaining a large percentage of genetic variation are not expected [1]. Consistently, the observed percentage of genetic variance explained by *HOG1* was 10.29 and 6.05% for aggressiveness and DON respectively. Similar results have been reported for *F. graminearum*, where genome-wide association reveled quantitative trait nucleotides explaining from 9% up to a maximum of 24% of genotypic variance [38]. The positive results of our study validate the application of candidate gene association mapping strategies to validate factors associated with pathogen aggressiveness under field conditions.

Isolates having the most frequent *HOG1* haplotype expressed on average the highest values for aggressiveness and DON production, which could be an effect of selection favoring these most aggressive genotypes. This fact highlights the importance of integrated plant disease management strategies to prevent undesired selection of the most aggressive genotypes of *F. culmorum*. Consistently, plant breeding must then steadily increase the level of resistance in cultivars, e.g. by pyramiding effective quantitative disease resistance loci.

### A suggested role of intronic regions in the expression of aggressiveness

The three SNPs found in *HOG1* are located in introns three and five. The fact that SNPs located within a noncoding region of *HOG1* are associated with the variation in aggressiveness and DON production could be explained by two hypotheses. Firstly, the detected SNPs are in linkage disequilibrium (LD) with causal polymorphisms in nearby genes or regulatory sequences that are responsible for the trait variance. Secondly, the nucleotide change within the intron is involved in post-transcriptional regulation, like alternative splicing, associated with the response to multiple stresses. Alternative splicing has been reported in fungi, including *F. verticillioides* [100] and *F. graminearum* [100–103]. In *F. graminearum*, grown under just one stress condition, 231 genes undergoing alternative splicing were found, but more genes are expected to follow post-transcriptional regulation if tested under different stresses [103], as shown for *Arabidopsis thaliana* [104]. Zhao et al. [103] demonstrated that for some genes in *F. graminearum* the alternative splicing events took place at different vegetative growth stages and suggested they might be important in adaptation of *F. graminearum* to changing environmental conditions. Furthermore, active functionality of intronic polymorphisms has been found in other organisms e.g. humans [105] and pigs [106].

Several SNPs in our study were discarded because of low calling rate, including some SNPs within *HOG1* located both in exons (positions 505, 609 and 686) and introns (positions 476, 484 and 730). On the other hand, some other genes had polymorphic SNPs with MAF > 5% but showed no association or had *p* values just slightly higher than the significance threshold (TRI147 and CUT563 + 3; Additional file 7). It must be noted, that this does not rule out a contribution of these genes to the variation in aggressiveness or DON production, as the population size of 100 isolates used in this study may have not allowed a statistically significant association.

## Conclusions

We phenotyped 100 *F. culmorum* isolates under field conditions and analyzed aggressiveness and DON production. Our results further support a quantitative pathogenicity model for the bread wheat - *F. culmorum* pathosystem. Variation in aggressiveness was largely explained by isolate genotype although the isolate-by-environment interaction was also significant, as expected for quantitative interactions. Of the 17 candidate genes ten showed polymorphisms that were tested for their association with the two traits. *HOG1* was identified as a component of the quantitative pathogenicity of *F. culmorum*. To the best of our knowledge, this is the first report of a protein kinase regulator explaining differences in field aggressiveness and mycotoxin production in a natural population of a plant pathogen.

## Additional files

Additional file 1: Number of isolates, sampling scheme, origin, and host characterizing the *F. culmorum* populations under study. (XLS 33 kb)

Additional file 2: Primer sequences, PCR conditions, and the full amplified sequences for the candidate genes used in this study. (XLS 53 kb)

Additional file 3: Best linear unbiased estimates (BLUEs) for mean aggressiveness and DON production calculated across four environments (location × year combinations) for 100 F. culmorum isolates. (XLSX 15 kb)

Additional file 4: Genotyping information of 100 isolates of F. culmorum. (XLSX 21 kb)

Additional file 5: Histograms for mean aggressiveness and DON production. Histograms of best linear unbiased estimates (BLUEs) for mean aggressiveness (top) and DON production (bottom) calculated across four environments (location × year combinations) for 100 *F. culmorum* isolates. (PDF 10 kb)

Additional file 6: Principal coordinate analysis for 100 *F. culmorum* isolates. Population structure and familial relatedness based on 10 SSR markers. Principal coordinate analysis for 100 *F. culmorum* isolates, based on modified Rogers’ distance. Number in parentheses refer to the proportion of variance explained by the principal coordinate. (PDF 17 kb)

Additional file 7: Characteristics of identified single nucleotide polymorphisms (SNPs) in the candidate genes and *p* values for association tests against mean aggressiveness and DON production. (LOG 402 bytes) (XLS 34 kb)

## Abbreviations

15-ADON: 15-acetyl-deoxynivalenol
3-ADON: 3-acetyl-deoxynivalenol
BH-MADR: Bonferroni–Holm with re-scaled MAD standardized residuals
BLUE: Best linear unbiased estimators
DON: Deoxynivalenol
FHB: Fusarium head blight
*h*^2^: Heritability
HOH: Hohenheim
LD: Linkage disequilibrium
MAF: Minor allele frequency
MAPK: Mitogen-activated protein kinase
NIV: Nivalenol
OLI: Oberer Lindenhof
PCoA: Principal coordinate analysis
REML: Restricted maximum likelihood
SNPs: Single nucleotide polymorphisms
SSRs: Single sequence repeats
ZEA: Zearalenone
