# Supplementary figures and images for: Candidate gene based association mapping in Fusarium culmorum for field quantitative pathogenicity and mycotoxin production in wheat
Source: BMC Genet. 2017 May 19;18:49. doi: 10.1186/s12863-017-0511-9 (PMC5438566; doi:10.1186/s12863-017-0511-9)

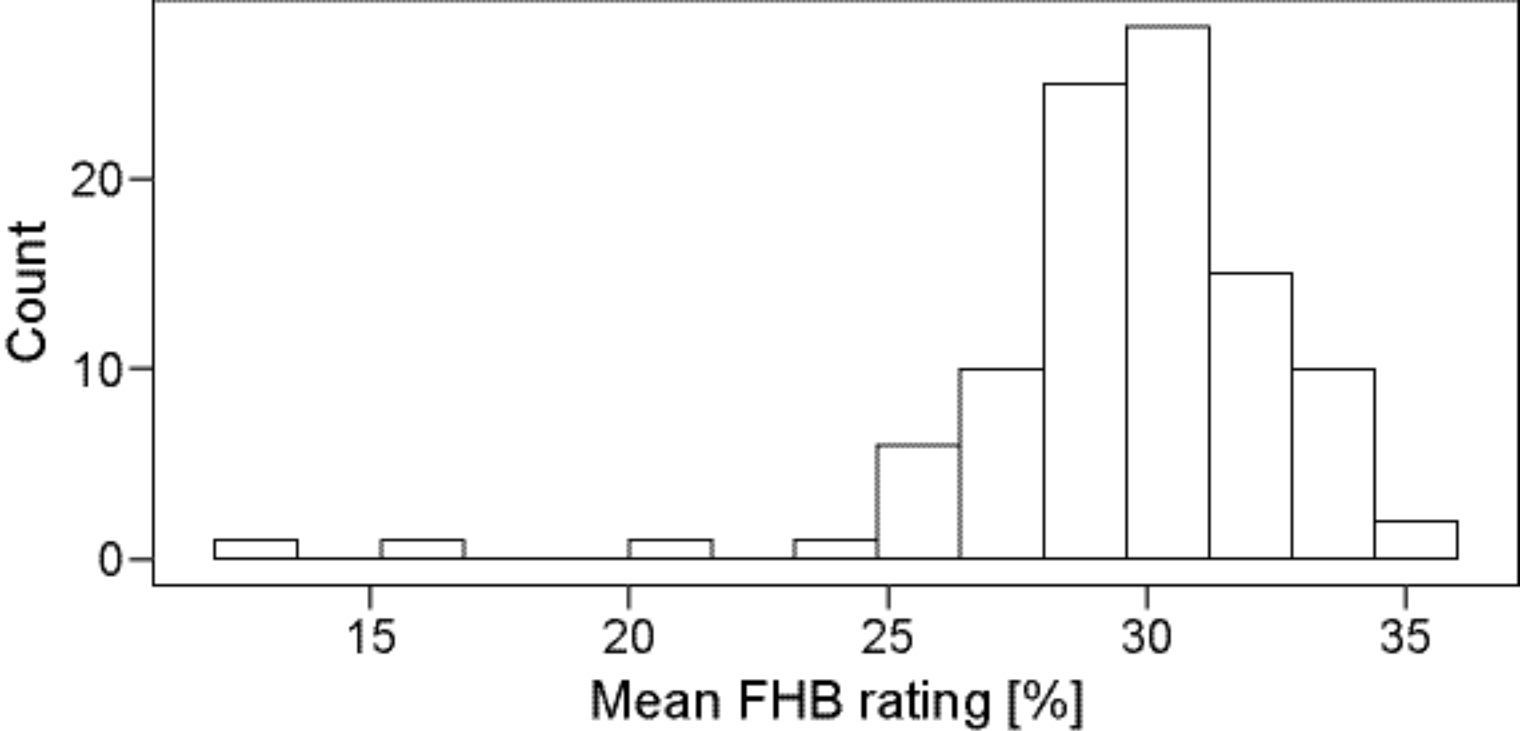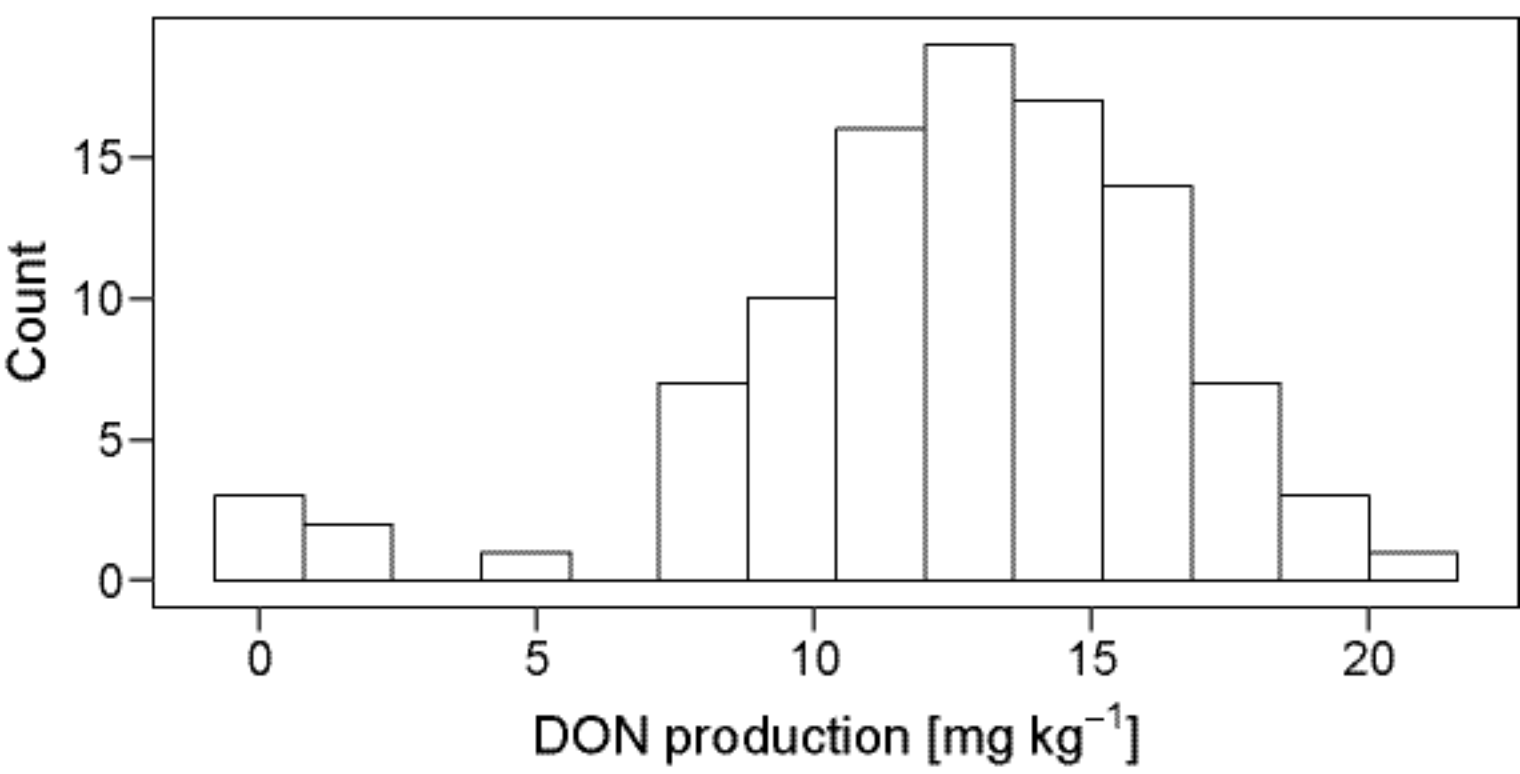

Supplement: Supplementary file 5 — Histograms for mean aggressiveness and DON production. Histograms of best linear unbiased estimates (BLUEs) for mean aggressiveness (top) and DON production (bottom) calculated across four environments (location × year combinations) for 100 F. culmorum isolates. (PDF 10 kb) [file 12863_2017_511_MOESM5_ESM.pdf]

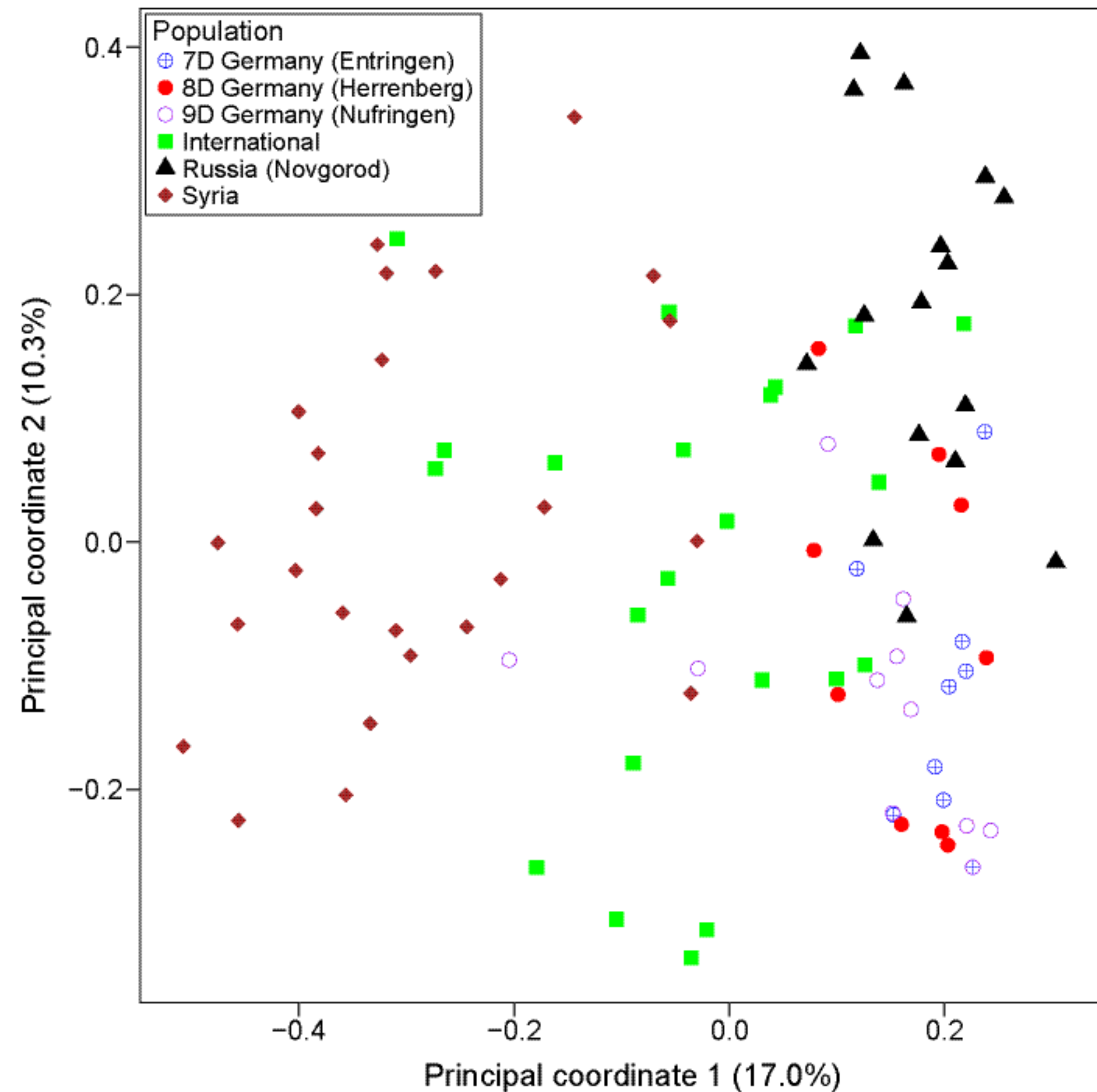

Supplement: Supplementary file 6 — Principal coordinate analysis for 100 F. culmorum isolates. Population structure and familial relatedness based on 10 SSR markers. Principal coordinate analysis for 100 F. culmorum isolates, based on modified Rogers’ distance. Number in parentheses refer to the proportion of variance explained by the principal coordinate. (PDF 17 kb) [file 12863_2017_511_MOESM6_ESM.pdf]
